# *Lactobacillus ruminis* Alleviates DSS-Induced Colitis by Inflammatory Cytokines and Gut Microbiota Modulation

**DOI:** 10.3390/foods10061349

**Published:** 2021-06-11

**Authors:** Bo Yang, Mingjie Li, Shuo Wang, R. Paul Ross, Catherine Stanton, Jianxin Zhao, Hao Zhang, Wei Chen

**Affiliations:** 1 State Key Laboratory of Food Science and Technology, Jiangnan University, Wuxi 214122, China; bo.yang@jiangnan.edu.cn (B.Y.); limingjie@stu.jiangnan.edu.cn (M.L.); ws87978797@163.com (S.W.); zhaojianxin@jiangnan.edu.cn (J.Z.); zhanghao61@jiangnan.edu.cn (H.Z.); 2 School of Food Science and Technology, Jiangnan University, Wuxi 214122, China; 3 International Joint Research Laboratory for Pharmabiotics & Antibiotic Resistance, Jiangnan University, Wuxi 214122, China; p.ross@ucc.ie (R.P.R.); catherine.stanton@teagasc.ie (C.S.); 4 APC Microbiome Ireland, University College Cork, T12K8AF Cork, Ireland; 5 Teagasc Food Research Centre, Moorepark, P61C996 Cork, Ireland; 6 National Engineering Research Center for Functional Food, Jiangnan University, Wuxi 214122, China; 7 Wuxi Translational Medicine Research Center, Jiangsu Translational Medicine Research Institute Wuxi Branch, Wuxi 214122, China

**Keywords:** *Lactobacillus ruminis*, DSS-induced colitis, inflammatory cytokines, SCFAs, gut microbiota

## Abstract

*Lactobacillus ruminis* can stimulate the immune response in vitro, but previous studies were only carried out in vitro and the anti-inflammatory effects of *L. ruminis* needs more in vivo evidences. In this study, the immune regulation and potential mechanisms of *L. ruminis* was investigated in DSS-induced colitis mice. *L. ruminis* FXJWS27L3 and *L. ruminis* FXJSW17L1 relieved the symptoms of colitis, including inhibition of colon shortening and colon tissue damage. *L. ruminis* FXJWS27L3 significantly reduced the pro-inflammatory cytokines IL-1β, TNF-α, and IL-17, while *L. ruminis* FXJSW17L1 significantly increased short chain fatty acids in mice feces. Moreover, *L. ruminis* FXJWS27L3 and *L. ruminis* FXJSW17L1 treatments significantly increased the gut microbiota diversity and balance the intestine microbiota profiles, which improved the imbalance of intestine microbiota composition to a certain extent. The results showed that *L. ruminis* can alleviate DSS-induced colitis, which possibly was related to promoting the expression of pro-inflammatory cytokines, up-regulating SCFAs and restoring the imbalance of gut microbiota.

## 1. Introduction

Ulcerative colitis (UC) is one of the inflammatory bowel diseases, with a high incidence and prevalence worldwide [1]. At present, routine therapy for most patients is to use 5-aminosalicylic acid (5-ASA) medicines (mesalazine, sulfasalazine), steroids or immunosuppressive agents to control inflammation [2], but severe side effects limit the usage of them [3]. Therefore, it is extremely important to find new treatment options for UC. Novel alternatives for IBD, such as prebiotics, probiotics, and monoclonal anti-TNF-α, are used instead of traditional therapies, which could rebalance the gut microbiota and modulate the immune response.

Numerous studies showed that a variety of *Lactobacillus*, especially *L. fermentum*, *L. reuteri*, *L. paracasei,* and *L. plantarum*, can relieve ulcerative colitis in animal model and clinical trials [4,5,6,7]. *L. ruminis* is one of the commensals in the gastrointestinal tract of humans and animals [8,9], and it persists throughout the life of the host. Currently, *L. ruminis* was reported to have pro-inflammatory effects in an in vitro study, in which *L. ruminis* ATCC25644, the type strain of the species, and its culture supernatant can activate the NF-κB pathway and increased IL-8 expression, which were mediated by TLR2 to a certain extent [10]. Another study also found that its flagellin can induce the production of interleukin-8 (IL-8) in human intestinal epithelial cell lines [11]. In addition, Taweechotipatr and colleagues found that *L. ruminis* exhibited the immunostimulatory property by activating the production of TNF-α in THP-1 monocyte [12]. However, the research on the immunomodulatory properties of *L. ruminis* is still insufficient. For example, only a few strains were studied and all those previous researches were only carried out in vitro, hence, the anti-inflammatory effects of *L. ruminis* need more investigations and evidences. Therefore, the study aimed to investigate the effects of *L. ruminis* on colitis in a DSS-induced mice model and the potential mechanism, and to provide a basis for further exploring the immunomodulatory properties of *L. ruminis*.

## 2. Materials and Methods

### 2.1. L. ruminis Culture Conditions

*L. ruminis* FXJWS27L3 and *L. ruminis* FXJSW17L1 were isolated from fecal samples of healthy volunteers in our previous work [13] and deposited at the Collection Center of Food Microbiology (CCFM), Jiangnan University. Both strains were cultured in de Man, Rogosa, and Sharpe medium (MRS) at 37 °C. Subsequently, the culture was centrifuged (8000× *g*, 20 min); the cell pellets was collected, washed three times with sterilized phosphate buffer solution (PBS, pH 7.4), concentrated in 30% (*v*/*v*) glycerol solution, and stored at −80 °C prior to use. For animal trial, the cell-pellet stock was washed twice with sterilized phosphate buffer solution (PBS, pH 7.4) and diluted to 5 × 10^9^ CFU/mL with 13% skim milk aqueous solution before preparing for gavage.

### 2.2. Animals and Experimental Design

The animal trial was approved by the Experimental Animal Management and Animal Welfare Ethics Committee of Jiangnan University (JN. No20191030c1041215(300)), and all methods were carried out in accordance with ARRIVE guidelines and regulations. C57BL/6J mice (male, 8-week-old) were purchased from GemPharmatech Co. Ltd., (Nanjing, China). All the animals were kept in Experimental Animal Center of Jiangnan University under standard conditions (constant temperature of 20 ± 2 °C, humidity of 50 ± 5%, and 12-h light-dark cycle).

A total of 40 mice were randomly divided into five groups (*n* = 8): control, DSS, mesalazine, FXJWS27L3 and FXJSW17L1 after one week of adaptation. The housing of mice in our research was carried out in compliance with the randomization on the ARRIVE guidelines. Four mice were randomly placed in each cage, and the treatment group was also randomly selected. Treatments were allocated based on online random number generators (https://www.graphpad.com/quickcalcs/randomize1/) (accessed on 15 December 2019), which was in accordance with the randomization principle. The calculation of sample size was based on law of diminishing return, which was called “resource equation” method [14]. By calculation, 8 mice each group were considered as enough sample size. The experimental period was 14-day totally and the experimental design was shown in Table 1. All the mice were free to sterilized water on day 1 to 7, and on day 8 to 14, except for control group, the mice in all the other groups were free to 2.5% (*w*/*v*) dextran sulphate sodium (DSS) solution. DSS solution was replaced every two days. From day 1 to 14, different composition (0.2 mL) was administered to each mouse once a day. 13% skim milk aqueous solution were intragastrically administered to the mice in control and DSS groups, mesalazine which was dissolved in 13% skim milk aqueous solution were intragastrically administered to mice in mesalazine group, and the mice in FXJWS27L3 and FXJSW17L1 group were gavaged with 5 × 10^9^ CFU/mL of *L. ruminis* FXJWS27L3 or *L. ruminis* FXJSW17L1, respectively.

After the mice were sacrificed, the colon tissues were isolated. The colon length of each mouse was recorded. Approximately 0.5 cm of the distal colon were taken and fixed in a 4% (*w*/*v*) paraformaldehyde, then, put the remaining colon in liquid nitrogen for quick freezing and store at −80 °C prior to use.

### 2.3. Assessment of Colitis

During DSS challenge, the weight loss, stool consistency, and hematochezia were measured at a fixed time every day, and the disease activity index (DAI) was calculated following the criteria [15,16]. The feces of each mouse were collected to observe the morphology, and the occult blood was measured by an Occult Blood Kit (Zhuhai Beisuo Biotechnology Co. Ltd., Zhuhai, China).

After the colon tissue was fixed, it was embedded in paraffin and stained with Hematoxylin and Eosin (H&E). Pathology section scanner was used to record the photomicrographs. The severity of colonic histological injury was scored from four perspectives: crypt damage, amount of inflammation, depth of inflammation, and the percentage involvement by the disease process following the Dieleman’s scoring system [17]. In tests, the numbers of each mouse were also random and discontinuous, and only colon tissues were provided to the raters without grouping information.

### 2.4. Determination of Cytokines in Colon

The colonic concentrations of IL-17, L-10, IL-4, IL-1β and TNF-α were determined by commercial ELISA kits (Shanghai Meilian Biotechnology Co. Ltd., Shanghai, China), and the BCA Protein Assay Kit (Beyotime Biotechnology, Shanghai, China) was used to measure the protein concentration by BCA method.

### 2.5. Immunofluorescence Staining of Colon

For immunofluorescence analysis, the cut sections were stained with 200 times dilution of ZO-1 (AB96587; Abcam, Cambs, UK), 100 times dilution of Claudin3 (AB15102; Abcam, Cambs, UK), and 100 times dilution of Occludin (AB216327; Abcam, Cambs, UK). All the antibodies were anti-rabbit. Slides were examined and analyzed using an epifluorescence microscope.

### 2.6. Determination of Short-Chain Fatty Acid Concentration and Gut Microbiota in Feces

The concentration of SCFA in feces was measured following the method described [18]. The FastDNA Spin Kit (MP Biomedicals, LLC, Irvine, CA, USA) was used to extract the genomic DNA. PCR amplification of the V3-V4 region of 16S rDNA was implemented, and the product was purified and quantified [19]. Library preparation, sequencing, and bioinformatic analysis were carried out on the basis of the previously described method [16]. Especially, some sequences need to be eliminated in the bioinformatic analysis, such as, sequences with a lower quality score (<30), a brief length (<200 bp), sequences containing equivocal bases, and appearing jumbles. Random rarefaction of each library was performed according to the sample with the least number of sequences. Sets of trimmed sequences with more than 97% identity were characterized as an operational taxonomic unit (OTU).

### 2.7. Statistical Analysis

GraphPad Prism 9.0 and SPSS26.0 were utilized for data analysis and plotting, and the significant difference was evaluated by one-way analysis of variance (ANOVA) followed by a Tukey test for multiple comparisons. The Shapiro-Wilk test and Kolmogorov-Smirnov test were used to test the normality of the data. If the within group distributions were not normally distributed, the significant difference was evaluated by Kruskal-Wallis test. If the variances were not equal, the significant difference was evaluated by Brown-Forsythe and Welch ANOVA followed by a Dunnett’s T3 test for multiple comparisons. The ultimate results were represented as the mean value ± standard deviation (SD). The significance is determined by the *p* value (*p* < 0.05). The confidence interval was 95% (95%CI). No data were lost and all mice provided all outcomes.

Briefly, the QIIME2 pipeline was used to analyze the sequence data. Alpha diversity (Shannon and Chao1 index) and beta diversity (weighted UniFrac distance) of gut microbiota were performed online Linear (https://www.microbiomeanalyst.ca/MicrobiomeAnalyst) (accessed on 1 March 2020) [19]. Linear discriminant analysis Effect Size (LEfSe) analyses were performed through online tools (https://huttenhower.sph.harvard.edu/galaxy) (accessed on 1 March 2020). Pearson correlation analysis of colonic SCFA concentration, significant genus, colitis indexes, and cytokines were performed by SPSS26.0.

## 3. Results

### 3.1. L. ruminis Relieved the Colitis Symptoms

**Figure 1:**
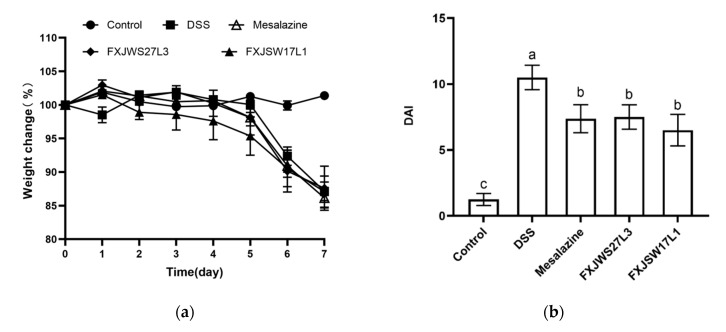

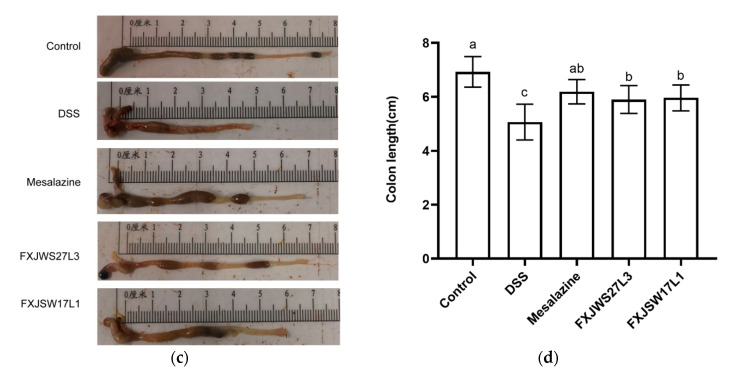
Effect of *L. ruminis* on the symptoms of colitis. (**a**) Body weight, (**b**) DAI, (**c**) Macroscopic pictures of colons, (**d**) Colon length. Letters a to c indicated statistically significant differences (*p* < 0.05). All data were presented as mean ± SD (*n* = 8 mice per group).

The effects of *L. ruminis* FXJWS27L3 and *L. ruminis* FXJSW17L1 on DSS-induced colitis symptoms were investigated and compared. During DSS exposure, the weight loss, stool consistency and hematochezia were measured every day. Mesalazine and *L. ruminis* treatments did not relieve the weight loss of mice (Figure 1a). On the 14th day, the DAI of mice in DSS group was as high as 10.50 ± 0.93, while that in mesalazine-treated mice was 7.38 ± 1.06, with a decrease of 29.7%; compared with DSS group, the treatment of *L. ruminis* FXJWS27L3 and *L. ruminis* FXJSW17L1 significantly reduced the DAI in mice with a decrease by 27.9% and 36.09%, respectively (Figure 1b). In control group, the colon was healthy light red, the feces were granular, and the length of the colon was 6.93 ± 0.62 cm; while the colon of mice in DSS group was dark red, the intestinal wall was swollen, and the intestinal cavity had obvious bloody contents, and the length of the colon was significantly decreased (5.07 ± 0.63 cm) (Figure 1c,d). The colon length of mice in mesalazine, FXJWS27L3 and FXJSW17L1 treated groups were 1.23, 1.16, and 1.18 times than that of the DSS-challenged mice, respectively (Figure 1d). The treatment of two *L. ruminis* strains could significantly alleviate the colon shortening caused by DSS exposure, which was as same as the result of DAI.

### 3.2. L. ruminis Reduced the Colonic Tissue Damage

**Figure 2:**
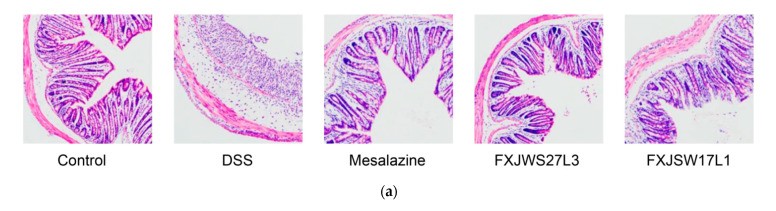

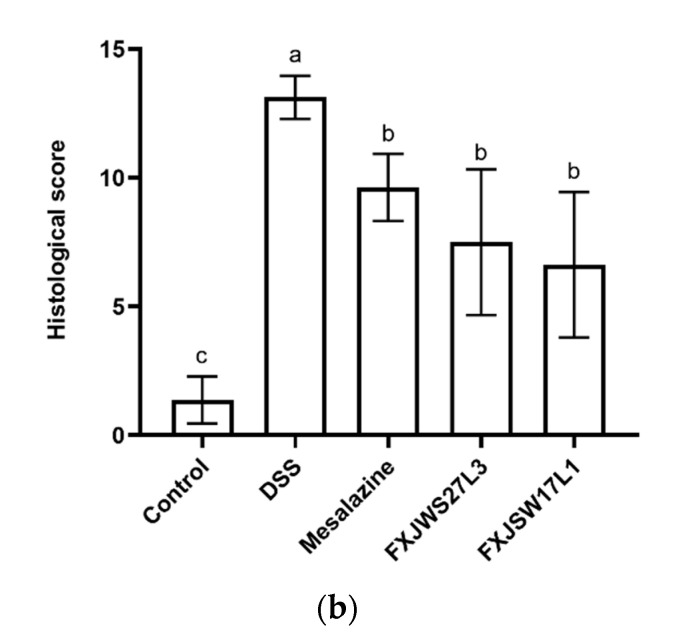
Effect of *L. ruminis* on histological injury. (**a**) Histological examination, Scale bars, 200 μm, (**b**) Colonic histological score. Letters a to c indicated statistically significant differences (*p* < 0.05). All data were presented as mean ± SD (*n* = 8 mice per group).

The colonic intestinal mucosa of the mice in control group was intact, with neat villi, healthy crypt structure and abundant goblet cells, without inflammatory cell infiltration (Figure 2a). However, the colon of DSS-exposed mice showed submucosal edema, severe inflammatory cell infiltration, complete disappearance of crypts and goblet cells, epithelial damage, and intestinal atrophy. The colonic histological score of mice in DSS group was 13.13 ± 0.83, which was 9.87 times than that of control group (Figure 2b). Whilst mesalazine, *L. ruminis* FXJWS27L3, and *L. ruminis* FXJSW17L1 treatments all significantly reduced the colonic histological score of mice compared with DSS group (Figure 2b). Additionally, in these three groups, the intestinal villi were relatively intact, the crypts partially disappeared, and the inflammatory infiltration was lighter.

### 3.3. L. ruminis Regulated the Inflammatory Cytokines

**Figure 3:**
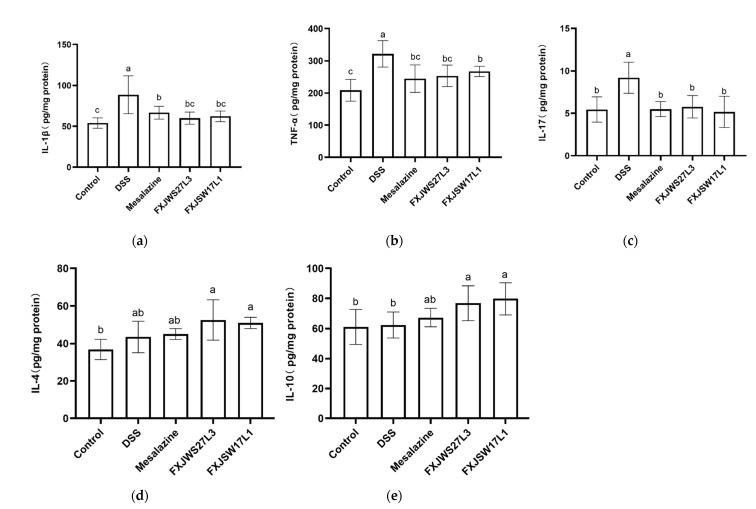
Effect of *L. ruminis* on cytokines. (**a**) IL-1β, (**b**) TNF-α, (**c**) IL-17, (**d**) IL-4, (**e**) IL-10. Letters a to c indicated statistically significant differences (*p* < 0.05). All data were presented as mean ± SD (*n* = 8 mice per group).

The anti-inflammatory cytokines (IL-4, IL-10) and pro-inflammatory cytokines (IL-1β, TNF-α, IL-17) in colon were measured to evaluate the modulation of *L. ruminis* on inflammatory cytokines. DSS exposure resulted in a significant increase in pro-inflammatory cytokines IL-17, TNF-α, and IL-1β in colon tissue, while the treatment of mesalazine significantly reduced those three pro-inflammatory cytokines (Figure 3a–c). IL-1β, TNF-α, and IL-17 in the *L. ruminis* FXJWS27L3-treated mice were significantly decreased by 32.18%, 21.08%, and 37.16%, respectively, compared with DSS group. In addition, the concentrations of IL-1β, TNF-α and IL-17 in *L. ruminis* FXJSW17L1-treated mice were also significantly reduced (Figure 3a–c). Additionally, compared with DSS group, mesalazine, *L. ruminis* FXJWS27L3, and *L. ruminis* FXJSW17L1 treatments had no significant effect on the IL-4 (Figure 3d,e), although there was a certain increasing trend.

### 3.4. L. ruminis Influenced the Tight Junction Protein in Intestinal Epithelial Cells

**Figure 4:**
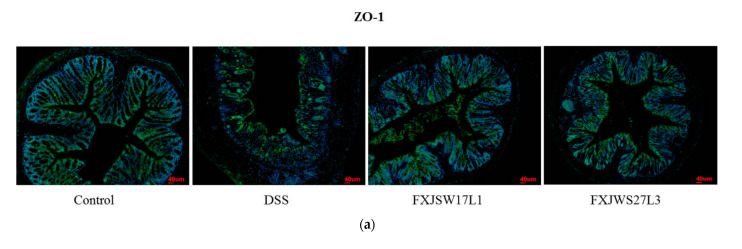

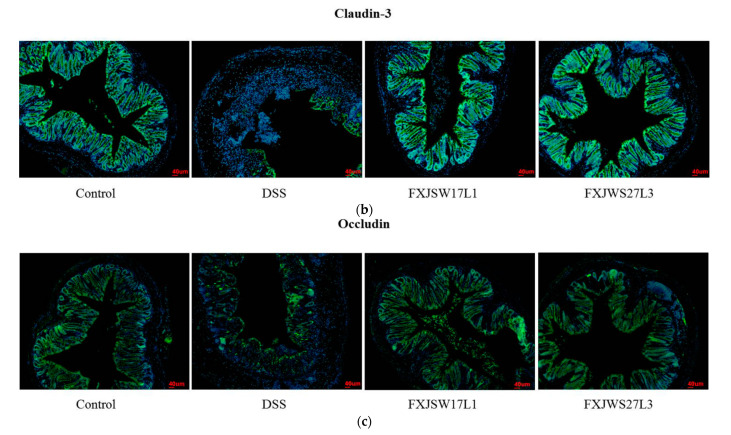
Effect of *L. ruminis* on TJ proteins in colon. (**a**) ZO-1, (**b**) Claudin-3, (**c**) Occludin.

In order to further verify the protective effect of *L. ruminis* on the intercellular tight junctions (TJ) proteins in the mice colon, the immunofluorescence was used to detect the location and content of the intercellular TJ proteins including ZO-1, Claudin-3, and Occludin. Under the reflected fluorescence illuminator, the cell nucleus showed blue fluorescence. Compared with the control group, the Occludin in DSS group was almost completely destroyed, the Claudin-3 and ZO-1 proteins were discontinuously distributed at the intestinal lumen edge, and the amount of these two proteins in the cell membrane and cytoplasm was dramatically reduced. Although *L. ruminis* FXJSW17L1 and *L. ruminis* FXJWS27L3 treatments retained the contents of Claudin-3 and Occludin integrally and had a relieving effect on the reduction in ZO-1 protein (Figure 4a–c). In addition, the results from immunofluorescence sections showed the similar changing tendency as the degree of colonic tissue damage.

### 3.5. L. ruminis Influenced the Concentration of SCFA in Feces

**Figure 5:**
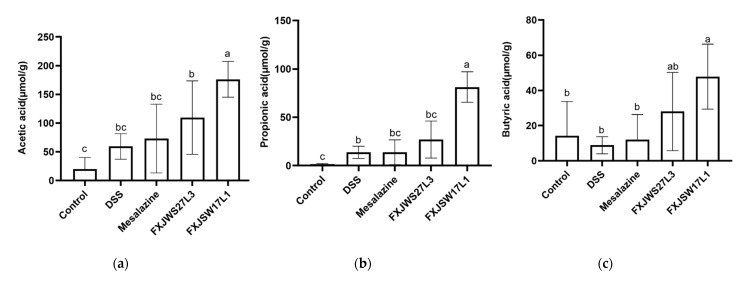

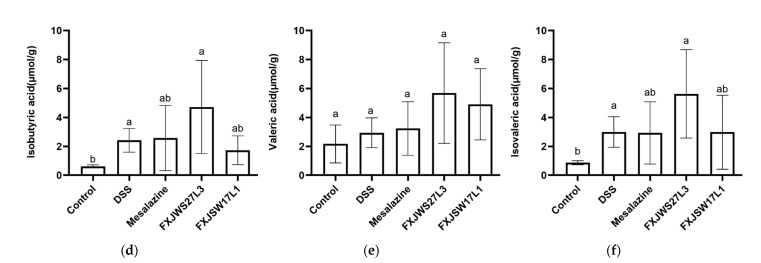
Effect of *L. ruminis* on SCFAs. (**a**) Acetic acid, (**b**) Propionic acid, (**c**) Butyric acid, (**d**) Isobutyric acid, (**e**) Valeric acid, (**f**) Isovaleric acid. Letters a to c indicated statistically significant differences (*p* < 0.05). All data were presented as mean ± SD (*n* = 8 mice per group).

The concentrations of SCFA in the feces after DSS challenge were measured, including acetic acid, propionic acid, butyric acid, isobutyric acid, valeric acid, and isovaleric acid. The results showed that, compared with control group, the concentration of SCFA in the DSS-exposed mice increased, except for butyric acid, although it was not significant (Figure 5a–f). *L. ruminis* FXJSW17L1 treatment significantly up-regulated the concentration of acetic acid, propionic acid, and butyric acid, which were 2.97, 5.90, and 5.39 times as much as DSS group; while the concentrations of isobutyric acid, valeric acid, and isovaleric acid did not show a significant change. All those six SCFAs in *L. ruminis* FXJWS27L3-treated mice feces showed an increasing trend as well, but there was no significant difference from DSS group.

### 3.6. The Modulation of L. ruminis on the Gut Microbiota Ruined by DSS

**Figure 6:**
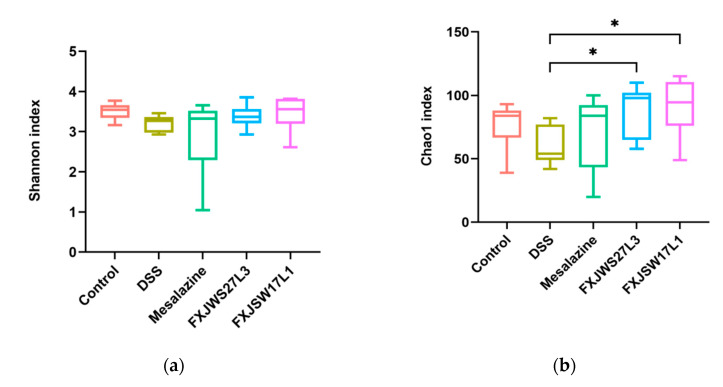

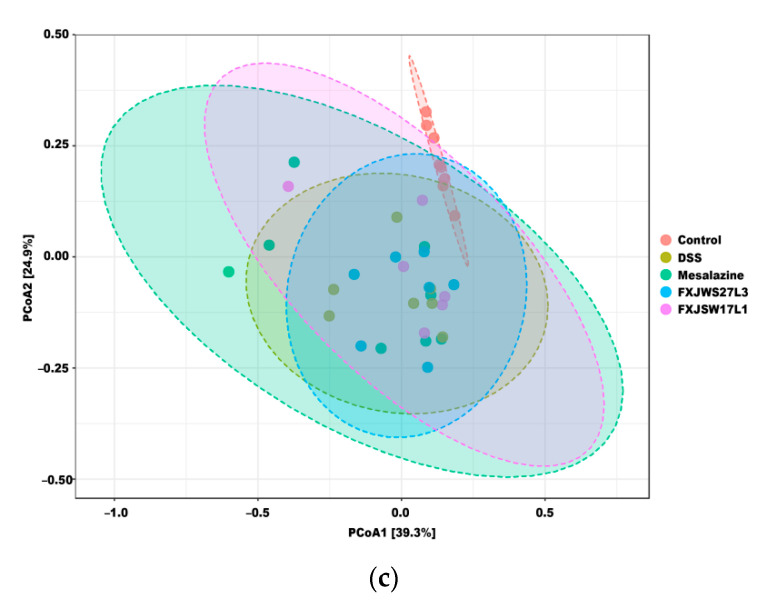
Effect of *L. ruminis* on the alpha and beta diversity of gut microbiota. (**a**) Shannon index, (**b**) Chao1 index, (**c**) Principal coordinates analysis (PCoA) of weighted UniFrac distances. * *p* < 0.05.

Alpha diversity was evaluated by Shannon and Chao1 indexes. Compared with DSS group, the Shannon index was not statistically different (Figure 6a), while Chao1 indexes of FXJWS27L3 group and FXJSW17L1 group were significantly increased (Figure 6b). The principal coordinates analysis (PCoA) of weighted UniFrac distance (*p* < 0.01) was used to reflect the beta diversity of gut microbiota. The results showed that there was a difference between gut microbiota of DSS-exposed mice and that of control group (Figure 6c). The treatment of *L. ruminis* FXJWS27L3 did not show a significant impact on the composition of gut microbiota, while the treatment of *L. ruminis* FXJSW17L1 caused a certain degree of movement of gut microbiota to control group, although it was not significant.

**Figure 7:**
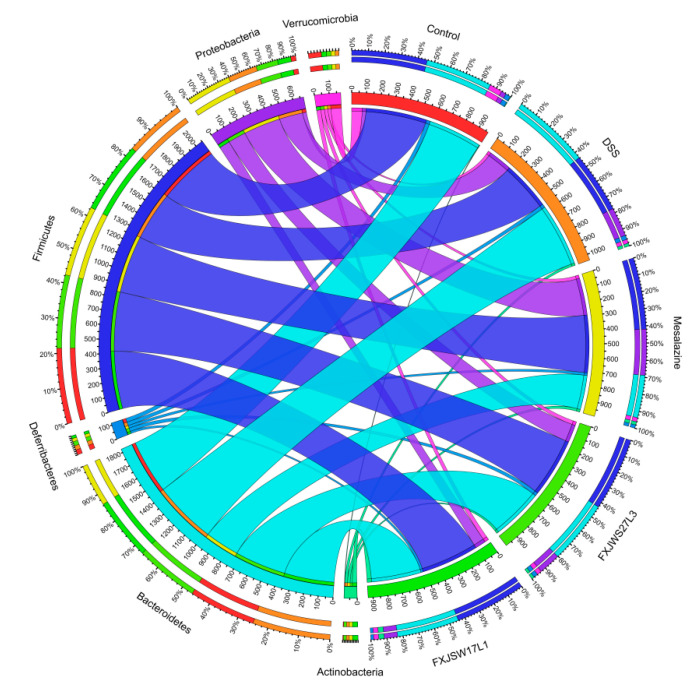
Microbial distribution at the phylum level. Different phylum has different colors, and the thickness of the lines represents the abundance of the species.

The relative abundance at the phylum level was analyzed. The relative abundance of Bacteroidetes and Proteobacteria increased in DSS group, while Firmicutes and Verrucomicrobia decreased. The relative abundance of Proteobacteria in *L. ruminis* FXJWS27L3- and *L. ruminis* FXJSW17L1-treated mice decreased compared with DSS group, although its relative abundance was higher than that in control group. Additionally, the relative abundance of Firmicutes increased in FXJWS27L3 group and FXJSW17L1 group compared with DSS group, while the relative abundance of Bacteroidetes and Proteobacteria decreased (Figure 7).

**Figure 8:**
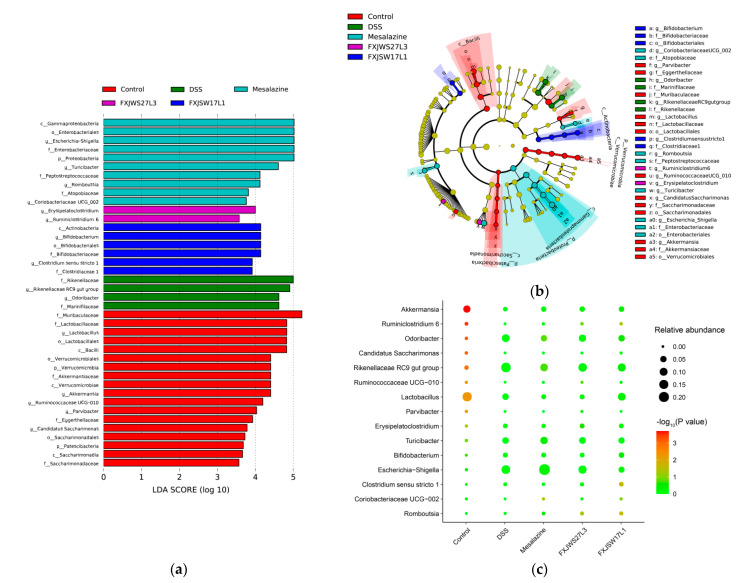
Effects of *L. ruminis* on dominant microorganisms. (**a**) Distribution histogram based on LDA, with a log LDA score above 3.0. The LDA histogram shows the species with significant differences, which are larger than the preset value. The color of the histogram represents each group, and the influence degree of the species with significant differences between different groups can be expressed according to the length of the histogram. (**b**) Cladogram based on LDA, with a log LDA score above 3.0. The circles radiating from the inside to the outside represent taxonomic levels from phylum to genus.The diameter of the small circles represents the relative abundance. (**c**) Relative abundance of the genus with significant differences in relative abundance between groups. All groups were compared with the DSS group.

To further analysis is the genus with significant differences in relative abundance among groups, the differences in the composition of gut microbiota among groups was analyzed by the LEfSe, and the LDA score histogram could identify statistically significant biomarkers and reveal the microbes with significant differences in relative abundance among groups. The DSS exposure led to significant increase in relative abundance of Rikenellaceae RC9 gut group and Odoribacter (*p* < 0.01), while the relative abundance of Ruminococcaceae UCG-010, Ruminiclostridium 6, *Akkermansia,* and *Lactobacillus* was significantly reduced (*p* < 0.01) (Figure 8a,b). The treatment of *L. ruminis* increased the abundance of *Lactobacillus*, *Akkermansia*, Ruminiclostridium 6, and Ruminococcaceae UCG-010. The differences between the two strains were that the relative abundance of *Lactobacillus* decreased in FXJWS27L3 group, which was increased in FXJSW17L1 group, although there was no significant difference (Figure 8c).

### 3.7. Correlation Analysis of Colitis Symptoms, Gut Microbiota, SCFA, and Cytokines

**Figure 9:**
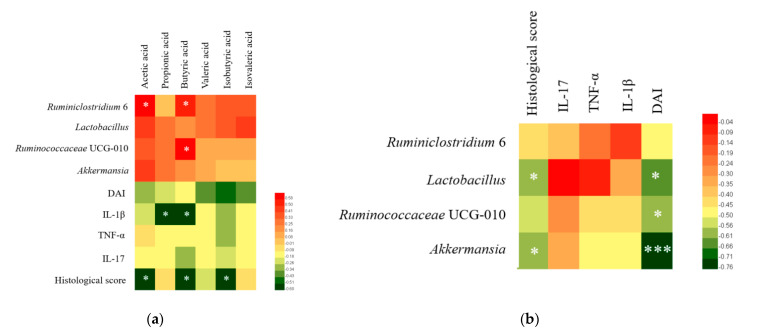
Correlation analysis of colitis symptoms, cytokines, SCFAs and gut microbiota. (**a**) Correaltion analysis of short-chain fatty acids with gut microbiota and colitis indicators, (**b**) Correlation analysis of gut microbiota with colitis indicators. * *p* < 0.05, *** *p* < 0.001.

Pearson correlation analysis was carried out among groups on the four genus with significant differences in abundance, SCFA, pro-inflammatory cytokines, DAI and histological score. The results showed that the concentrations of six SCFAs were positively correlated with Ruminococcaceae UCG-010, Ruminiclostridium 6, *Akkermansia,* and *Lactobacillus* (Figure 9a). In addition, there was a significant positive correlation between Ruminiclostridium 6 and the concentrations of acetic acid and butyric acid, and a significant positive correlation between Ruminococcaceae UCG-010 and the concentration of butyric acid (*p* < 0.05). The concentrations of SCFAs were negatively correlated with DAI, pro-inflammatory cytokines and histological score (Figure 9a). Ruminococcaceae UCG-010, Ruminiclostridium 6, *Akkermansia,* and *Lactobacillus* were all negatively correlated with DAI, histological score, and pro-inflammatory cytokines (Figure 9b).

## 4. Discussion

Studies have shown that *L. ruminis* can stimulate cells to produce pro-inflammatory cytokines in vitro and had potential immune regulation functions, but its in vivo benefit is still unclear. Therefore, in this study, the immune regulation of *L. ruminis* FXJWS27L3 and *L. ruminis* FXJSW17L1 was investigated through the DSS-induced colitis in mice. Both *L. ruminis* FXJWS27L3 and *L. ruminis* FXJSW17L1 can alleviate the symptoms of colitis (including DAI and colon shortening) in mice. The results of colonic histopathological scores showed that both *L. ruminis* strains can reduce the damage of epithelial structure and submucosal edema, the infiltration of inflammatory cells and the disappearance of crypts caused by DSS exposure.

Abnormal intestinal immune response is one of the characteristics of UC [20,21], and TNF-α and IL-1β are pro-inflammatory mediators caused by the immune response of colitis [22]. TNF-α can cause mucosal inflammation and intestinal barrier injury and is a key factor in inducing inflammatory bowel disease. TNF-α can activate the NF-κB pathway and further induce the expression of TNF-α and other pro-inflammatory cytokines such as IL-1β [23]. IL-17 produced by Th17 cells has the ability to promote the production of a variety of inflammatory cytokines and is an important part of the pathogenesis of colitis [24]. In this study, the changes of anti-inflammatory cytokines IL-4 and IL-10 in colon were evaluated. DSS exposure resulted in a large amount of expression of the pro-inflammatory cytokines in colon, including IL-17, TNF-α, and IL-1β, and the treatment of *L. ruminis* FXJWS27L3 and *L. ruminis* FXJSW17L1 significantly inhibited the increase in pro-inflammatory cytokines. As for the anti-inflammatory cytokines IL-4 and IL-10, *L. ruminis* treatment caused an upward trend compared with DSS group, although it was not statistically significant. Therefore, our results showed that *L. ruminis* down-regulated the pro-inflammatory cytokines, and up-regulated the anti-inflammatory cytokines. In another study on *Lactobacillus* alleviating DSS-induced colitis, it was also found that the treatment of *L. plantarum* AR113 and *L. casei* AR342 can significantly down-regulate the concentrations of TNF-α and IL-1β in mice colon tissues and up-regulate the anti-inflammatory cytokine IL-10 expression, and, thus, played a role in alleviating colitis [4]. Additionally, *L. paracasei*, *L. fermentum* and *L. reuteri* have been shown to significantly reduce the IL-17 as well [5,6,7].

In addition, it was found in previous in vitro experiments that *L. ruminis* can induce the expression of pro-inflammatory cytokines TNF-α and IL-8 [10,11,12], and in the current work, *L. ruminis* FXJWS27L3 and *L. ruminis* FXJSW17L1 can down-regulate the pro-inflammatory cytokines to exert its anti-inflammatory effect on DSS-induced colitis. The results indicated that the direction of immune regulation of *L. ruminis* might be strain-dependent. Similar results were found in the study of *L. plantarum* immune regulation [25], in which *L. plantarum* CCFM8610 had a significant inhibitory effect on the production of pro-inflammatory cytokines IL-1β, IL-17F and TNF-α, while *L. plantarum* CCFM382 did not show any obvious inhibitory effects.

Tight junctions (TJ), as a crucial part of the physical barrier of the intestines, are a skeleton to connect cells and maintain the normal physiological functions of selective permeability. Among various TJ proteins, intramembrane protein ZO-1, the transmembrane protein Claudin-3 and Occludin are representative [26]. The previous studies have shown that diverse *Lactobacillus* strains have different effects on the content and localization of TJ proteins, containing ZO-1, Claudin-3 and Occludin [26]. Moreover, the respites of *Lactobacillus* on colitis are related to its recovery of TJ proteins [27]. On the basis of that, protection on the intestinal barriers may be an important factor for *L. ruminis* to relieve colitis as well.

Short-chain fatty acids are metabolites released when gut microbiota digest the dietary fiber in the intestine, and mainly include acetic acid, propionic acid, butyric acid, valeric acid, isobutyric acid, and isovaleric acid. It has been reported in the literature that SCFAs were able to protect intestinal barrier function [28,29] and participate in the regulatory mechanism of host intestinal immunity [30]. Increasing the intake of SCFA was effective in the treatment of colitis [31]. Acetate and propionate can stimulate GPR43 to up-regulate the expression of regulatory T cell transcription factors (Foxp3), and cause the proliferation of colonic regulatory T cells (Treg), thereby promoting the production of the anti-inflammatory cytokine IL-10 [32]. Butyric acid can increase the anti-inflammatory ability of macrophages and dendritic cells (DC) by stimulating GPR109A, promoting Treg differentiation and down-regulating the expression of inflammatory factor IL-17 [33]. In this study, the treatment of *L. ruminis* FXJSW17L1 significantly up-regulated the concentration of acetic acid, propionic acid and butyric acid in feces, and the concentration of valeric acid and isovaleric acid showed an upward trend as well; and the six SCFAs in *L. ruminis* FXJWS27L3-treated mice feces also showed an upward trend. The results of correlation analysis also showed that all those six SCFAs were negatively correlated with DAI, pro-inflammatory cytokines, and histological scores. A variety of *Lactobacillus* has been shown to improve colitis by regulating the production of SCFAs. For example, the treatment of *L. acidophilus* up-regulated the concentration of butyric acid and propionic acid, thereby, significantly protecting the intestinal tract of mice with colitis [34]. *L. casei* LH23 was able to up-regulate the concentration of acetic acid, propionic acid, and butyric acid to compensate for the reduction in SCFAs caused by DSS exposure [35]. In addition, *Lactococcus lactis* ML2018 alleviated colitis by promoting the production of SCFAs in the intestine [36]. Therefore, regulating the production of SCFAs may be one of the key ways for *L. ruminis* to relieve colitis.

Some previous studies have shown that inflammatory bowel disease can reduce the diversity of gut microbiota, change the composition of gut microbiota and lead to the destruction of the intestinal micro-ecology [37,38]. The imbalance of gut microbiota plays a vital role in the pathogenesis of UC [39,40,41]. In this study, treatment of *L. ruminis* FXJWS27L3 and *L. ruminis* FXJSW17L1 significantly increased the species richness of gut microbiota, and the results of beta diversity analysis showed that gut microbiota of mice in *L. ruminis* FXJSW17L1 group shifted to a certain extent to control group. Similar results were found in previous study on *L. fermentum* alleviating colitis, in which the Chao1 index was significantly increased compared with DSS group, and the disturbance caused by DSS treatment in the gut microbiota was less severe when the mice were administered with *L. fermentum* [5].

It has been reported that the increased relative abundance of Proteobacteria can be used as a microbial biomarker for gut microbiota imbalance [42]. *L. ruminis* FXJWS27L3 and *L. ruminis* FXJSW17L1 can inhibit the increase in relative abundance of Proteobacteria caused by DSS exposure, and restore the gut microbiota homeostasis of mice to a certain extent. DSS-induced colitis can lead to a decrease in Firmicutes and an increase in Bacteroidetes in the gut microbiota of mice [43], and Firmicutes has been reported with anti-inflammatory effects [44]. In our study, compared with DSS group, the relative abundance of Firmicutes in FXJWS27L3 group and FXJSW17L1 group increased, while the relative abundance of Bacteroidetes decreased, thereby improving the imbalance of Firmicutes and Bacteroidetes and the microbiota structure. In the studies of other *Lactobacillus*, it was also found that *L. paracasei* treatment reduced the increase in the abundance of Bacteroidetes caused by DSS treatment [6], and the treatment of *L. reuteri* I5007 caused a significantly increased relative abundance of Firmicutes and a decreased Proteobacteria relative abundance [7].

At the genus level, *L. ruminis* FXJWS27L3 and *L. ruminis* FXJSW17L1 treatments increased the relative abundance of *Akkermania*, Ruminiclostridium 6, and Ruminococcaceae UCG-010. *Akkermansia* is related to intestinal immunity and plays a key role in intestinal homeostasis [45]. Previous studies found that *L. fermentum* KBL374 and *L. fermentum* KBL375 can alleviate DSS-induced colitis, which was related to the significant increase in *Akkermansia* and other beneficial bacteria in the gut microbiota [5]. *Ruminiclostridium* can degrade the polysaccharides to generate acetate and butyrate, thereby promoting the development of the immune system [46]. Ruminococcaceae is related to the production of butyric acid, and previous research reported that this genus had the anti-inflammatory activity [47]. The correlation analysis in our study also showed that there was a significant positive correlation between Ruminiclostridium 6 and the concentrations of acetic acid and butyric acid, and a significant positive correlation between Ruminococcaceae UCG-010 and the concentration of butyric acid. Additionally, Ruminococcaceae UCG-010, Ruminiclostridium 6 and *Akkermania* were negatively correlated with DAI, histological score and pro-inflammatory cytokines. Therefore, *L. ruminis* FXJWS27L3 and *L. ruminis* FXJSW17L1 improved the imbalance of gut microbiota in DSS-induce colitis mice to some extent, which may be another important factor for *L. ruminis* to alleviate colitis.

## 5. Conclusions

*L. ruminis* FXJSW17L1 and *L. ruminis* FXJWS27L3 can alleviate DSS-induced colitis in mice. The effective patterns mainly include decreasing the pro-inflammatory cytokines (TNF-α, IL-1β, IL-17), up-regulating the SCFAs, especially acetic acid, propionic acid, and butyric acid, and restoring the imbalance of gut microbiota. This study firstly confirms the anti-inflammatory effects of *L. ruminis* in vivo, which will provide a reference for further exploring the immune regulation function of the species.

## 6. Patents

Bo Yang, Wei Chen, Shuo Wang, et al. A *Lactobacillus ruminis* strain alleviating colitis and use thereof [P]. Chinese patent, 2020116254075.

Wei Chen, Bo Yang, Shuo Wang, et al. A *Lactobacillus ruminis* strain protecting intestinal barrier and use thereof [P]. Chinese patent, 2020116327362.

## Figures and Tables

**Table 1:** Animal model experimental design.

Group	Daily Gavage Treatment (0.2 mL)	1–7 Day	8–14 Day
Control	13% skim milk aqueous solution	Free drinking sterilized water	Free drinking sterilized water
DSS	13% skim milk aqueous solution	Free drinking sterilized water	Free drinking DSS solution (2.5%)
Mesalazine	10 mg/mL mesalazine	Free drinking sterilized water	Free drinking DSS solution (2.5%)
FXJWS27L3	5 × 10^9^ CFU/mL *L. ruminis* FXJWS27L3	Free drinking sterilized water	Free drinking DSS solution (2.5%)
FXJSW17L1	5 × 10^9^ CFU/mL *L. ruminis* FXJSW17L1	Free drinking sterilized water	Free drinking DSS solution (2.5%)

## Data Availability

Data sharing not applicable.
